# Comparative Transcriptome Analysis Provides Insights into the Polyunsaturated Fatty Acid Synthesis Regulation of *Fat-1* Transgenic Sheep

**DOI:** 10.3390/ijms21031121

**Published:** 2020-02-07

**Authors:** Rongsong Luo, Zhong Zheng, Chunrong Yang, Xiaoran Zhang, Lei Cheng, Guanghua Su, Chunling Bai, Guangpeng Li

**Affiliations:** 1 State Key Laboratory of Reproductive Regulation & Breeding of Grassland Livestock, School of Life Sciences, Inner Mongolia University, Hohhot 010070, China; lrs0729@163.com (R.L.); zhengzhong@imu.edu.cn (Z.Z.); zxrnmgdx@163.com (X.Z.); chenglei19840920@163.com (L.C.); guanghuasu@imu.edu.cn (G.S.); gpengli@imu.edu.cn (G.L.); 2 College of Animal Science and Technology, Huazhong Agricultural University, Wuhan, Hubei 430070, China; chryang@mail.hzau.edu.cn; 3 College of Innovation Technology, Inner Mongolia University, Hohhot 010070, China

**Keywords:** Comparative transcriptome, *Fat-1*, Transgenic animal, PUFAs, *FADS2*

## Abstract

Transgenic technology has huge application potential in agriculture and medical fields, such as producing new livestock varieties with new valuable features and xenotransplantation. However, how an exogenous gene affects the host animal’s gene regulation networks and their health status is still poorly understood. In the current study, *Fat-1* transgenic sheep were generated, and the tissues from 100-day abnormal (DAF_1) and normal (DAF_2) fetuses, postnatal lambs (DAF_4), transgenic-silencing (DAFG5), and -expressing (DAFG6) skin cells were collected and subjected to transcriptome sequencing, and their gene expression profiles were compared in multiple dimensions. The results were as follows. For DAF_1, its abnormal development was caused by pathogen invasion but not the introduction of the *Fat-1* gene. *Fat-1* expression down-regulated the genes related to the cell cycle; the NF-κB signaling pathway and the PI3K/Akt signaling pathway were down-regulated, and the PUFAs (polyunsaturated fatty acids) biosynthesis pathway was shifted toward the biosynthesis of high-level *n*-3 LC-PUFAs (long-chain PUFAs). Four key node genes, *FADS2*, *PPARA*, *PRKACA*, and *ACACA,* were found to be responsible for the gene expression profile shift from the *Fat-1* transgenic 100-day fetus to postnatal lamb, and *FADS2* may play a key role in the accumulation of *n*-3 LC-PUFAs in *Fat-1* transgenic sheep muscle. Our study provides new insights into the FUFAs synthesis regulation in *Fat-1* transgenic animals.

## 1. Introduction

Transgenic animals can be defined as animals in which new or altered genes have been experimentally inserted into their genome by genetic engineering techniques [1]. The world’s first transgenic mouse was generated with a broad-spectrum expression construct of the herpesvirus thymidine kinase gene driven by the SV40 promoter through pronuclear injection in 1980 [2]. Then Wilmut I. and colleagues took the lead in producing the world’s first somatic cell cloned sheep, Dolly, in 1997, which pioneered a new era of mammalian somatic cell cloning, bridged the gap between animal cell manipulation and individual manipulation, and also injected new vitality into animal transgenic technology research [3]. The combination of transgenic technology and SCNT (somatic cell nuclear transfer) technology paved the way for the birth of transgenic cloned livestock [4,5]. In 2003, Brophy et al. increased the copy number of the β-casein gene (*CSN2*) and κ-casein gene (*CSN3*) in the bovine genome by transgenic cloning technology and obtained cattle that could produce milk with higher levels of β-casein and κ-casein [6]. In 2004, Kuroiwa reported the production of transgenic cattle unsusceptible to mad cow disease [7]. These studies demonstrated the power of transgenic cloning technologies in the producing of new livestock varieties with great new features and high economic values.

However, new questions also emerged along with the development of transgenic cloning technologies, such as whether these animals are as healthy as their none-transgenic counterparts, how does the exogenous gene work in these animals, how can we improve exogenous gene expression efficiency in host animals, are these animals or their products safe as human food? Until now, GMOs (genetically modified organisms) are still under strict regulations around the world, and no transgenic livestock is allowed to be industrialized. Thus, more basic research should be done to answer the above-mentioned questions.

The *n*-3 polyunsaturated fatty acids (PUFAs), such as α-linolenic acid (ALA, 18:3n−3), docosahexaenoic acid (DHA, 22:6n−3), and eicosapentaenoic acid (EPA, 20:5n−3), have important physiological functions and play important roles in cell proliferation, cell differentiation, and immunity. However, mammals cannot naturally produce *n*-3 PUFAs by themselves and must rely on dietary supply [8]. The fatty acid desaturase-1 gene *Fat-1* is an unsaturated fatty acid dehydrogenase gene of *Caenorhabditis elegans*, which can convert *n*-6 fatty acids to *n*-3 fatty acids by adding a double bond to the unsaturated fatty-acid hydrocarbon chain [9]. *Fat-1* transgenic mice had a higher amount of *n*-3 and lower amount of *n*-6 fatty acids in their organs and tissues without dietary *n*-3 when compared to non-transgenic mice [10]. *Fat-1* transgenic pigs were also rich in *n*-3 PUFAs [11], providing the possibility of creating livestock with high *n*-3 PUFAs level and high potential economic values. In the year 2011, our group produced *Fat-1* transgenic cattle with increased *n*-3 PUFAs and decreased *n*-6 PUFAs in their tissues and milk [12].

Long-chain PUFAs (LC-PUFAs) are PUFAs with twenty or more carbon atoms, which can further be divided into *n*-6 LC-PUFAs such as arachidonic acid (ARA, 20:4n−6) and adrenic acid (ADA, 22:4*n*−6) and *n*-3 LC-PUFAs such as EPA, docosapentaenoic acid (DPA, 22:5n−3), and DHA [13]. In particular, EPA and DHA have well recognized beneficial roles in retinal and brain development during infancy and the prevention of autoimmune disorders and some types of cancers and cardiovascular diseases [14,15]. The two essential short chain dietary 18C-PUFAs, ALA and linoleic acid (LA, 18:2n−6), are the key substrates for the biosynthesis of *n*-3 and *n*-6 PUFAs, respectively. Firstly, in the presence of two desaturation enzymes encoded by fatty acid desaturase 2 and 1 (*FADS2* and *FADS1*) and one elongation enzyme encoded by *ELOVL5*, EPA and ARA were synthesized from ALA and LA, respectively. Thereafter, with another elongation enzyme encoded by *ELVOL 5/2*, DPA and ADA could be generated from EPA and ARA, respectively. Finally, DHA can be generated from DPA with a Δ-4 desaturation enzyme also encoded by *FADS2*. Moreover, DHA could also be generated from EPA with three additional biosynthetic steps [16,17]. Since the biosynthesis of *n*-3 LC-PUFAs and *n*-6 LC-PUFAs shares the same enzymatic steps, their corresponding 18C-PUFAs, ALA and LA, and metabolic intermediates compete with each other in the liver and other tissues [18]. However, the overall capacity of converting 18C-PUFAs to LC-PUFAs in the human body is limited [19]. A dramatic increase in LA ingestion (mainly from vegetable oil) in modern life breaks the balance and shifts the pathway toward the biosynthesis of high levels of *n*-6 LC-PUFAs and away from *n*-3 LC-PUFAs, which has become a risk factor for western-type cancers, cardiovascular and cerebrovascular diseases, and also for allergic hyper-reactivity [20,21].

Sheep is the major livestock widely kept in northern and western China, and the sheep husbandry industry contributes to the main part to the economy of the pastoral areas in these places. Sheep that produce mutton with high *n*-3 PUFAs contents may benefit people’s health and have high economic values. In the year 2012, our team produced *Fat-1* transgenic sheep with sheep fetal fibroblasts transfected with the pCMV-fat-1-IREs-EGFP plasmid and pCAG-fat-1-IREs-EGFP plasmid as donor cells [22,23]. In the current study, in order to reveal the expression status of the exogenous *Fat-1* gene and how it affects PUFA synthesis in transgenic fetuses and lambs, we collected tissue samples from abnormal and normal transgenic fetuses on the 100th gestational day, postnatal lambs, as well as transgenic silencing and expressing skin cells, to conduct transcriptome sequencing. The gene expression profiles of these samples were compared in multiple dimensions, and the mechanisms of how *Fat-1* affects the biosynthesis of *n*-3 LC-PUFAs were discussed.

## 2. Results

### 2.1. Generation of Fat-1 Transgenic Fetuses and Lambs

**Figure 1:**
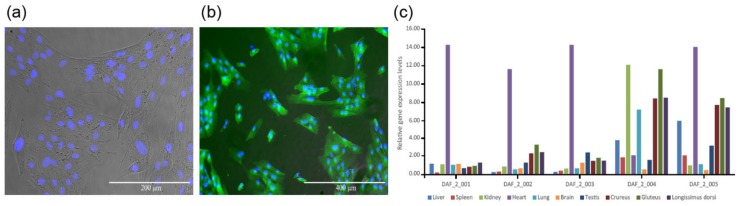
*Fat-1* transgenic positive cell line and detection of *Fat-1* gene expression levels by q-PCR. (**a**) Merged picture of *Fat-1* transgenic cells under an optical microscope and a fluorescent microscope, stained with Hoechst 33342 to show the nucleus. (**b**) Merged picture of *Fat-1* transgenic positive cells under a fluorescent microscope with strong EGFP fluorescence (**c**) The gene expression levels of exogenous *Fat-1* in different tissues of *Fat-1* transgenic fetuses. The expression level of *Fat-1* in DAF_2_001 liver tissue was considered to be 1.

The expression vector pCAG-*fat-1*-IREs-EGFP was used to transfect sheep fetal fibroblasts, and positively transfected cell lines were selected (Figure 1a,b). Reconstructed embryos were produced with these cells as donor cells, and the in vitro cultured blastocysts were transferred into the uterus of estrus synchronized surrogates by surgery. Finally, twelve ewes successfully got pregnant. At the time of 100 gestational days, six fetuses were recovered from recipient ewes through cesarean. One of them (DAF_1) was abnormally developed, and the rest five were normal (DAF_2_001 to DAF_2_005). The remaining recipient ewes delivered six lambs (DAF_4_001 to DAF_4_006). Ten different tissues from the liver, spleen, kidney, heart, lung, brain, testis, crureus, gluteus, and dorsal muscle of each normal fetus were collected to check the exogenous *Fat-1* expression by qPCR. The results showed that all these fetuses expressed *Fat-1* in their tissues (Figure 1c, Table S1), which indicated a successful transgenic animal production workflow.

### 2.2. Global Gene Expression Profiles of Different Tissue and Skin Cell Samples

Five different gluteus tissues from an abnormal fetus (DAF_1_001 to DAF_1_005), gluteus tissues from five normal fetuses (DAF_2_001 to DAF_2_005), gluteus tissues from six postnatal lambs (DAF_4_001 to DAF_4_006), and transgenic-silencing (DAFG5_001 to DAFG5_006) and -expressing lamb skin cells (DAFG6_001 to DAFG6_006) obtained through flow cytometry by EGFP fluorescent intensity were collected and subjected to transcriptome sequencing (Table S2). A total of 203,926,778 paired-end reads 150 bp in length were obtained. The average Q30 value was above 94%, and the mapping rate of each sample was above 80% (Table S3). The gene expression levels for each sample were estimated. As shown in Table S4, around 22% of 21,158 annotated genes were expressed above an FPKM of 100, about 11% between 50–100 FPKM, 19% between 10–50 FPKM, and the rest under 10 FPKM. To ensure the reliability of the gene expression for further analysis, a pair-wise correlation between any two biological replicates in each group was checked based on the normalized FPKM values. The correlation coefficients were all above 0.95, suggesting a high level of reproducibility and rationality of sample collection (Figure S1).

**Figure 2:**
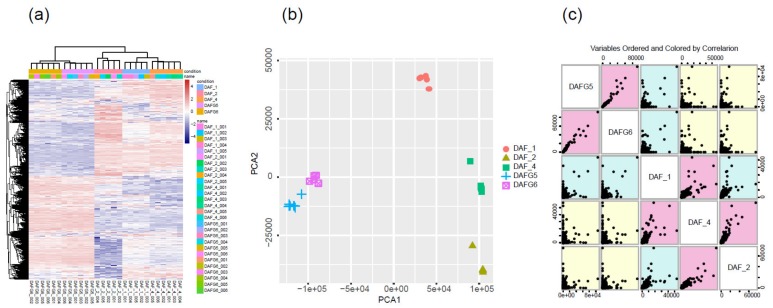
Schematic illustration of global gene expression profiles and sample reproducibility of three different gluteus tissue resources (abnormal fetus and normal fetuses at 100 gestational days, postnatal lambs) and two cell types (transgenic-silencing and -expressing cells). (**a**) Heatmap comparing global transcriptome profiles of different samples; the pink color represents a higher expression level, and the blue color represents a lower expression level. (**b**) PCA illustrated the reproducibility and rationality of sample collection. (**c**) Variable order of sample pairs colored by correlation.

To determine the primary patterns of gene expression, the hierarchical clustering analysis of all expressed genes was conducted based on their similar expression modulations. For the global gene expression pattern, the transcriptome profiles across different samples showed that DAFG5 and DAFG6 had similar expression patterns, while DAF_1, DAF_2, and DAF_4 clustered together (Figure 2a). To further test the relationships among samples, PCA was conducted using the FPKM values of reference genes for all samples. We observed that the different data sets from the same sample types clustered together. Three gluteus tissue types (DAF_1, DAF_2, DAF_4) were clearly separated from each other and distinguished from the two cell types (DAFG5 and DAFG6). As expected, DAF_1, which from the abnormal fetus, was distinguished from DAF_2 and DAF_4, while DAF_2 and DAF_4 were much closer (Figure 2b). The scatter plot of variable orders of sample pairs and correlation color also identified two distinct segmentations, gluteus tissues and cell samples (Figure 2c). These results suggested that these samples were highly reproducible, and great differences existed between the three gluteus tissues and two cell samples at the transcriptome level.

### 2.3. Gene Expression Differences between Abnormal and Normal Fetuses

**Figure 3:**
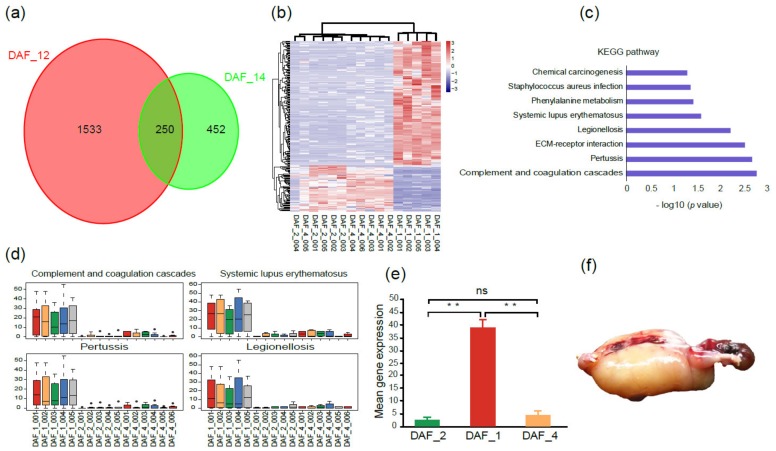
Gene expression patterns and KEGG analysis between abnormal and normal fetuses. (**a**) Venn diagram shows the overlapped DEGs between DAF_1 and DAF_2 and between DAF_1 and DAF_4. (**b**) Heatmap of overlapped DEGs between abnormal and normal fetuses; the pink color represents a higher expression level, and the blue color represents a lower expression level. (**c**) KEGG enriched pathways of overlapped DEGs. (**d**) Comparison of gene expression levels of the genes within four disease and pathogen-associated pathways between abnormal and normal fetuses. (**e**) Genes related to pathogen and disease infections had significantly higher expression levels in DAF_1 than in DAF_2 or DAF_4 (*t*-test, ** *p* < 0.01, ns *p* > 0.05). (**f**) The abnormal fetus DAF_1 had a typical edema phenomenon.

Since one cloned fetus (DAF_1) stopped developing at 100 gestational days, to find out the reason, a comparative transcriptome analysis was carried out among this fetus, other normal fetuses (DAF_2), and postnatal lambs (DAF_4). A total of 1783 DEGs (differentially expressed genes) were obtained between DAF_1 and DAF_2, and 702 DEGs between DAF_1 and DAF_4 (log2 (fold change) > 2, adjusted *p*-value ≤ 0.05, Figure 3a), which indicated that large differences existed between abnormal and normal fetuses. Then, the 250 overlapped DEGs were extracted and analyzed. The heatmap showed DAF_2 and DAF_4 had closer gene expression profiles, while DAF_1 had a clearly different one (Figure 3b).

To gain insights into the categories and functions of these genes which may potentially be associated with abnormal fetal development, KEGG enrichment analysis of these 250 DEGs was conducted. Pathways including legionellosis and staphylococcus aureus infection, pertussis and systemic lupus erythematosus and complement and coagulation cascades, and phenylalanine metabolism were enriched, which mainly belong to the categories of pathogen and disease invasion and immune response (Figure 3c). The expression levels of the genes within these pathways were all significantly higher in DAF_1 than in DAF_2 and DAF_4 (Figure 3d,e). Genes such as C7, IL1A, MYD88, and TLR5 were highlighted in these pathways, which have been reported to be involved in the innate immunity and inflammatory response [24,25,26,27,28,29]. Furthermore, at the time of sample collection, the abnormal fetus showed a typical edema phenomenon of pathogen infection (Figure 3f). These results indicated that the abnormal development of DAF_1 was caused by pathogen invasion in the uterus, but not the introduction of the exogenous *Fat-1* gene.

### 2.4. Gene Expression Differences between Fat-1 Transgenic-Silencing and -Expressing Skin Cells

**Figure 4:**
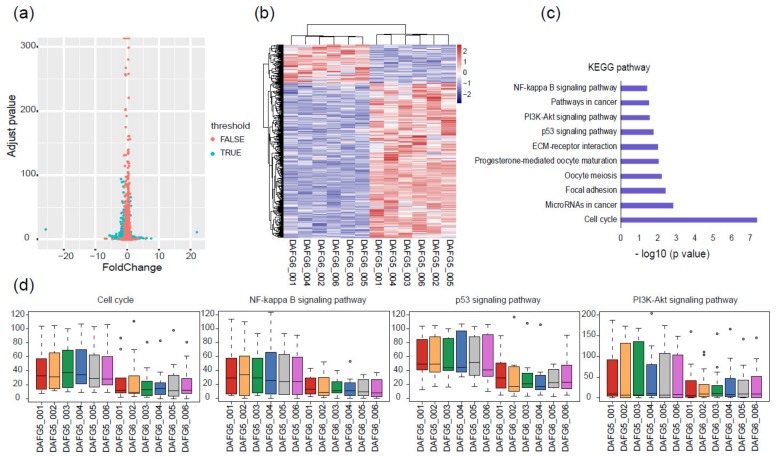
Different gene expression profiles of *Fat-1* transgenic-silencing and -expressing skin cells. (**a**) Volcano plot showing 466 DEGs between *Fat-1* transgenic-silencing and -expressing cells. (**b**) Heatmap of DEGs between transgenic-silencing and -expressing cells; the pink color represents a higher expression level, and the blue color represents a lower expression level. (**c**) KEGG pathway analysis showed enriched pathways were mainly related to cell proliferation and immunity. (**d**) Relative expression levels of the genes within four typical KEGG pathways; circles are outliers.

Transgenic-silencing cells accounted for a small part in the skin of *Fat-1* transgenic sheep, providing an ideal model to analyze the effect of exogenous *Fat-1* on the global gene expression profiles of the cells within individuals. Therefore, comparative transcriptome analysis was conducted on the FACS-sorted transgenic-silencing (DAFG5) and -expressing (DAFG6) lamb skin cells. A total of 466 DEGs were identified, of which 373 were up-regulated in DAFG5, and 93 were up-regulated in DAFG6 (Figure 4a,b). Genes related to cell proliferation and cellular immunity were found in these DEGs, including *CDK1,* which is involved in the regulation of the cell cycle, *PDGFB,* which is involved in cell proliferation and migration, and *ATM*, *MAP3K7*, and *LTB,* which are involved in cellular immunity [30].

To comprehensively characterize the transcriptomic difference and further uncover the most representative and correlated functional pathways between these two cell types, KEGG enrichment analysis was conducted based on the above-mentioned 466 DEGs. The cell cycle, *NF-κB* signaling pathway, *p53* signaling pathway, and *PI3K/Akt* signaling pathway were among the main pathways (Figure 4c). Genes belonging to these four pathways generally had higher expression levels in DAFG5 than in DAFG6 (Figure 4d), suggesting a possible suppression effect of *Fat-1* expression on these genes in transgenic-expressing cells.

### 2.5. Gene Expression Differences between Fat-1 Transgenic Fetuses and Postnatal Lambs

**Figure 5:**
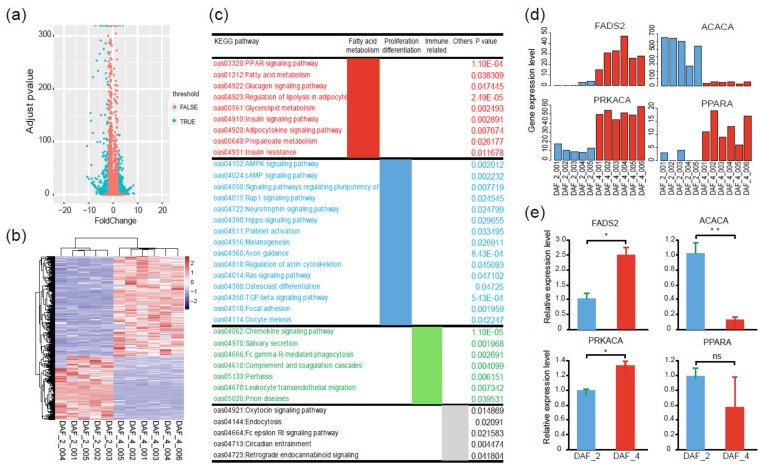
Different gene expression profiles between *Fat-1* transgenic 100-day fetuses and postnatal lambs. (**a**) Volcano plot of 1447 DEGs between *Fat-1* transgenic 100-day fetuses (DAF_2) and postnatal lambs (DAF_4). (**b**) Heatmap showing different transcription levels of DEGs between DAF_2 and DAF_4; the pink color represents a higher expression level, and the blue color represents a lower expression level. (**c**) KEGG pathway analysis of the DEGs between DAF_2 and DAF_4. (**d**) Comparison of four key node gene expression levels between DAF_2 and DAF_4 according to transcriptome data. (**e**) Gene expression validation of four key node genes by qPCR (*t*-test, ** *p* < 0.01, * *p* < 0.05, ns *p* > 0.05).

To gain more insights into the biosynthesis and metabolism of PUFAs at the gene expression level, a comparative transcriptome analysis was conducted between DAF_2 and DAF_4. A total of 1447 DEGs were identified, of which 560 DEGs were up-regulated in DAF_2 and 887 DEGs were up-regulated in DAF_4 (Figure 5a,b), indicating a gene expression shift from the 100-day fetus to postnatal lamb. The KEGG analysis provided 36 significantly enriched signaling pathways, which could be divided into three groups. Group I was mainly related to fatty acid biosynthesis and metabolism, including the PPAR signaling pathway, fatty acid metabolism signaling pathway, and regulation of lipolysis in the adipocyte signaling pathway. Group II was mainly related to cell proliferation, differentiation, and apoptosis, including the AMPK signaling pathway, TGF-beta signaling pathway, Hippo signaling pathway, and Ras signaling pathway. Group III was mainly related to the regulation of disease and the immune system (Figure 5c). A previous study showed that inflammatory and innate immune processes may also be coordinated and regulated by lipid metabolism [31]. These results indicated that many physiological processes associated with fatty acid metabolism were different between the 100-day fetus and postnatal lamb.

**Figure 6:**
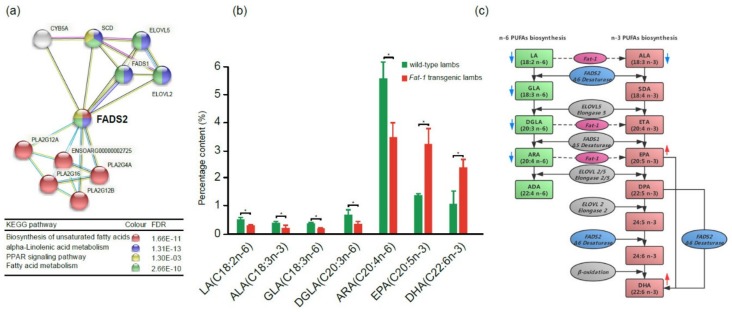
*FADS2* in the PUFA biosynthesis-related PPI network and roles of *Fat-1* and *FADS2* in *n*-6 and *n*-3 LC-PUFA (long-chain polyunsaturated fatty acids) biosynthesis pathways. (**a**) The *FADS2* gene is involved in four key pathways related to biosynthesis and metabolism of PUFAs. Different colored balls represent different signaling pathways. (**b**) The contents of *n*-3 and *n*-6 PUFAs in the gluteus tissues of *Fat-1* transgenic lambs and wild type lambs (t-test, *p < 0.05). (**c**) Diagram of *n*-6 and *n*-3 PUFAs biosynthesis pathways and roles of *Fat-1* and *FADS2* in the pathways, modified from F. H. Chilton et al. 2017 [17]. The red arrow or blue arrow beside each PUFA means its content increased or decreased, respectively, in *Fat-1* transgenic sheep when compared to wild type sheep.

To further narrow and target the key regulatory factors, protein and protein interaction network (PPI) analysis was conducted with the above-mentioned DEGs. The results demonstrated that these DEGs were mainly related to fatty acid metabolism, cell proliferation and differentiation, and immunity (Figure S2). It is worth noting that several DEGs were highlighted as node genes in the PPI network, especially *FADS2*, *PPARA*, *PRKACA*, and *ACACA* (Figure 6a, Figure S3). Among these node genes, *FADS2* acts as a fatty acyl-coenzyme A (CoA) desaturase that introduces a cis double bond at the carbon 6 of the fatty acyl chain [32]. *PPARA* is a ligand-activated transcription factor, which regulates the peroxisomal beta-oxidation pathway of fatty acids [33]. *ACACA* is involved in the biosynthesis of long-chain fatty acids by playing a key role in the conversion of acetyl-CoA to malonyl-CoA [34]. According to the transcriptome data, gene expression levels of *FADS2*, *PPARA,* and *PRKACA* were up-regulated in postnatal lambs, while the gene expression level of *ACACA* was down-regulated (Figure 5d). To confirm the credibility of the transcriptome data, the gene expression levels of above-mentioned four genes were compared between DAF_2 and DAF_4 by qPCR. As expected, the relative expression levels of *FADS2*, *ACACA,* and *PRKACA* had the same trend as the transcriptome data, while *PPARA* had a different one (Figure 5e, Table S5). These results indicated that the transcriptome data were reliable.

### 2.6. PUFA Contents between 100-day Fat-1 Transgenic Fetuses and Wild Type Fetuses

In order to evaluate the effect of *Fat-1* on the PUFA contents of transgenic sheep, the gluteus tissues from 100-day transgenic fetuses and 100-day wild type fetuses were collected and subjected to GC-MS to compare their PUFA compositions. The results showed that the percentages of those PUFAs in the *n*-6 PUFAs biosynthesis pathway, including LA, γ-linolenic acid (GLA, 18:3n-6), dihomo-γ-linolenic acid (DGLA, 20:3n-6), and ARA, were significantly reduced in transgenic fetuses, while for the PUFAs in the *n*-3 PUFAs biosynthesis pathway, *n*-3 LC-PUFAs EPA and DHA were significantly increased. (Figure 6b,c). The results confirmed that the *n*-3 LC-PUFAs, which have great healthy benefits to the human body, significantly accumulated in *Fat-1* transgenic sheep.

## 3. Discussion

Transgenic animals, including rabbits, pigs, goats, sheep, and cattle, have been produced in the past decades [12,35,36,37,38]. However, the activity and impact of exogenous genes in host animals, as well as their biosafety, are among the topics of most concern. In the current study, we produced *Fat-1* transgenic sheep and compared the transcriptome of normal and abnormal fetuses, transgenic-silencing and -expressing skin cells, 100-day fetuses, and postnatal lambs to gain a better understanding of how the exogenous *Fat-1* gene affects the gene expression profiles of transgenic sheep, as well as the PUFA biosynthesis networks.

Abnormal fetal development and early death were the common issues the researchers faced during the production of cloned animals or transgenic cloned animals [39]. In our case, one abnormal fetus was found, with development stopped in the recipient’s uterus when we recovered the 100-day transgenic fetuses from the surrogates. To find out the reason, we conducted comparative transcriptome analysis between this abnormal fetus and five normal ones. Through the KEGG analysis of their DEGs, pathways mainly related to pathogen infection and immune response were enriched, such as staphylococcus aureus, tuberculosis, leishmaniasis, pertussis, and complement and coagulation cascades. The immune function between the mother and the fetus can be partially reflected by the maternal circulation system, in which an immune-suppressive response to the fetus occurs. Among the highlighted genes, C7 plays a key role in the innate and adaptive immune response by forming pores in the plasma membrane of target cells [24]. IL1A (interleukin-1α), which is produced by activated macrophages, stimulates thymocyte proliferation by inducing IL-2 release, B-cell maturation, and proliferation, as well as fibroblast growth factor activity [29]. MYD88 is an adapter protein involved in the Toll-like receptor and IL-1 receptor signaling pathway in the innate immune response. TLR5 (Toll-Like Receptor 5) locates on the cell surface and participates in the activation of innate immunity and inflammatory response [27]. Upon ligand binding such as bacterial flagellins, TLR5 recruits intracellular adapter proteins MYD88 and TRIF, leading to NF-κB activation, cytokine secretion, and induction of the inflammatory response [25]. No fatty acid biosynthesis and metabolism-related pathways were enriched; based on this and the typical edema phenomenon of the dead fetus, we believed that its abnormal development was caused by pathogen invasion and not the introduction of *Fat-1*.

The expression of exogenous genes in transgenic animals is the prerequisite of subsequent gene expression network research. In our study, the *Fat-1* gene was randomly inserted into the sheep genome, and its expression was driven by the CAG promoter. The monoclonal transgenic positive cell lines were used to reconstruct cloned embryos. However, the transgenic fetuses possessed both *Fat-1* expressing and silencing cells in their tissues. Our previous study indicated that the hypermethylation status in the specific CAG promoter region may be responsible for preventing *Fat-1* expression in transgenic-silencing cells [23]. In the current study, the skin cells with or without *Fat-1* expression were further compared at the transcriptome level. We found pathways related to cell proliferation, cell differentiation, and apoptosis, and the immune response were enriched based on their DEGs. In *Fat-1* transgenic-expressing cells, the genes related to the cell cycle showed lower expression levels. In our previous study, ESCs derived from *fad3b* (has similar functions as *Fat-1* [40]) transgenic mice showed a much slower cell cycle with an increased *n*-3 PUFA content in the cells [41]. In other studies, *n*-3 PUFAs could also inhibit the proliferation of different kinds of cancer cells by regulating their cell cycle [42,43,44,45]. Therefore, *Fat-1* expression cells may also have a lower proliferation rate than *Fat-1* silencing cells or cells in wild type sheep.

The introduction of the *Fat-1* gene in mouse, pig, sheep, cattle, and cow resulted in high levels of *n*-3 PUFAs and significantly lower *n*-6/*n*-3 PUFA ratios in their tissues [10,11,12,23,46,47]. In our study, the same results were obtained by comparing *n*-6 and *n*-3 PUFA contents between 100-day transgenic fetuses and wild type fetuses. As shown in Figure 6c, *n*-6 and *n*-3 PUFA biosynthesis pathways shared the same enzymatic steps and physiologically balance in animal or human bodies. However, a high initial LA level brought about by increased vegetable consumption could shift the balance toward *n*-6 LC-PUFA synthesis and result in insufficient *n*-3 LC-PUFAs in humans due to the limitation of the total LC-PUFA synthesis capacity. In the current study, the initial ALA and LA levels in 100-day transgenic sheep and wild type sheep were considered to be the same since all the pregnant ewes were housed under the same condition. However, in *Fat-1* transgenic sheep, the amounts of *n*-6 PUFAs (LA, GLA, DGLA, and ARA) were significantly reduced while the amounts of *n*-3 LC-PUFAs (EPA and DHA) were significantly increased. Given the fact that Fat-1 in *C. elegans* could convert LA to ALA, DGLA to ETA, and ARA to EPA [9], we believe that the *Fat-1* gene driven by the CAG promoter functioned well in transgenic sheep and shifted PUFA biosynthesis balance toward *n*-3 PUFA biosynthesis. High *n*-3 LC-PUFA contents in the muscle make the *Fat-1* transgenic sheep a potential n-3 PUFA food resource.

In the comparison of gene expression profiles between transgenic-silencing and -expressing skin cells, 466 DEGs were found. In the main pathways derived from these DEGs, the pathways related to the cell cycle, NF-κB, p53, and PI3K/Akt were down-regulated in transgenic-expressing skin cells. *n*-3 PUFAs are closely involved in immune regulation, anti-inflammatory activity, cell proliferation, and cell differentiation [48]. A high content of *n*-3 PUFAs or a low ratio of *n*-6/*n*-3 PUFAs could down-regulate the activity of NF-κB and inhibit tumor formation [49]. It was also reported that *n*-3 LC-PUFAs EPA and DHA could inhibit myogenesis and down-regulate the muscle-related gene expression, therefore down-regulating the PI3K/Akt pathway [50]. Moreover, DHA could also suppress LPS-mediated PI3K/Akt and NF-κB activation in mouse kidney [51]. Therefore, with the introduction of *Fat-1* genes, high intracellular *n*-3 LC-PUFA levels delivered profound changes to the host animal related to cell proliferation and differentiation, inflammatory, as well as tumor formation. It will be interesting and important to conduct a lifetime tracking on the growth rate, health status, and life span of *Fat-1* transgenic sheep, as well as other *Fat-1* transgenic livestock, to provide important references for humans under the condition of high ALA or *n*-3 LC-PUFA intake. Several reports indicated that *n*-3 PUFAs had an anticancer effect by activating the p53 pathway [52,53,54,55]. However, in our case, the p53 signaling pathway was also down-regulated in transgenic-expressing cells. Due to the limited available information, the underlying mechanism needs further investigation.

In the current study, we compared the gene expression profile between *Fat-1* transgenic 100-day fetuses and postnatal lambs. KEGG analysis from 1447 DEGs showed a broad gene expression pattern shift from fetuses to lambs. The differences were mainly related to fatty acid biosynthesis and metabolism, cell proliferation, differentiation, and apoptosis, as well as the regulation of disease and the immune system. PPI analysis yielded the same results. Among the key node genes, *FADS2* and *PRKACA* were up-regulated in postnatal lambs according to transcriptome and qPCR data. *FADS2* plays a central role in the biosynthesis of *n*-3 LC-PUFAs. The product of *FADS2* catalyzes the conversion from LA to GLA and ALA to stearidonic acid (SDA, 18:4n-3) in lower eukaryotes, nematodes, and plants, and ADA to tetracosapentaenoic acid (24:5n-6), 9,12,15,18,21-tetracosapentaenoic acid (24:5n-3) to 9,12,15,18,21-tetracosapentaenoic (24:6n-3) and DPA to DHA in mammals [32,56,57]. In *Fat-1* transgenic sheep, after Fat-1 increased the metabolic intermediates ETA and EPA in the *n*-3 LC-PUFA biosynthesis pathway, FADS2 may catalyze the production of the final *n*-3 LC-PUFA DHA and make DHA accumulate to a high level. Since higher *FADS2* activity was found in postnatal lambs than in 100-day fetuses, the DHA production efficiency would be even higher during individual development.

## 4. Materials and Methods

### 4.1. Ethical Statement

All animal work was conducted in accordance with the Guide for the Care and Use of Laboratory Animals and the Chinese Veterinary Medical Association (CVMA), approved by the Institutional Animal Care and Use Committee at Inner Mongolia University (IMU-2015-09, 8/6/2015), which is fully accredited by Association for Assessment and Accreditation of Laboratory Animal Care International. The experiments complied with the Chinese Code of Practice for the Care and Use of Animals for Scientific Purposes, including conditions for animal welfare and handling prior to sacrifice.

### 4.2. Generation of Fat-1 Transgenic Lambs and Sample Collection for RNA-seq

Sheep (*Ovis aries*) fetal fibroblasts were transfected with the expression vector pCAG-*fat-1*-IREs-EGFP, and positive cell lines were obtained by G418 (geneticin 418) and green fluorescent screening. The transgenic positive cells were used as the donor cells to reconstruct SCNT embryos. Then, the cloned embryos were cultured in vitro until the blastocyst stage and were transferred into the uterus of estrous synchronized surrogates by surgery. At the time of 100 gestational days, transgenic fetuses were obtained by cesarean, and the gluteus tissues of each fetus (five normal fetuses and one abnormal fetus) were collected for RNA-seq. Ten different tissues (from liver, spleen, kidney, heart, lung, brain, testis, crureus, gluteus, and dorsal muscle) of each fetus were collected to detect exogenous *Fat-1* gene expression. To compare the gene expression profiles of transgenic-silencing and -expressing cells in postnatal lambs, the skin sample was digested into single cells and separated by flow cytometry according to their fluorescent intensity. Then, the transgenic-silencing and -expressing cell samples were subjected to RNA-seq. Gluteus tissues from six postnatal lambs were also collected for RNA-seq. Finally, a total of 28 samples including the gluteus of 100-day abnormal fetus (DAF_1), 100-day normal fetuses (DAF_2), postnatal lambs (DAF_4), and *Fat-1* transgenic-silencing (DAFG5) and -expressing (DAFG6) skin cells were collected to conduct transcriptome sequencing.

### 4.3. RNA Isolation, Library Construction, and Transcriptome Sequencing

Total RNA of each sample was extracted with TRIzol (Invitrogen, USA) according to manufacturer’s instructions. The extracts were then fractionated on 1% agarose gels to monitor RNA contamination and degradation, followed by assessment of RNA yield with the Qubit^®^ RNA Assay Kit in a Qubit^®^ 2.0 Fluorometer (Life Technologies, CA, USA). The purity and integrity of RNA were checked with the NanoPhotometer^®^ spectrophotometer (IMPLEN, CA, USA) and Bioanalyzer 2100 (Agilent Technologies, CA, USA) with RIN number > 6.8. Poly(A) mRNA isolated from about 3 μg of total RNA with poly-T oligo-attached magnetic beads (Invitrogen) was cleaved into shorter fragments using a metal catalyst at elevated temperature. The cleaved RNA fragments were then reverse transcribed using Invitrogen DNA Polymerase I and RNase H (New England Biolabs) to generate first-strand cDNA and second-strand cDNA. The short cDNA fragments generated were purified, end-repaired, poly-adenylated, and ligated to indexed sequencing adaptors. To selectively enrich library fragments ranging from 150 to 200 bp in length, the ligated cDNA molecules were purified with the AMPure XP system (Beckman Coulter, Beverly, USA) and used as templates for PCR amplification based on the results of agarose gel electrophoresis. After gel purification, the amplified PCR products were subjected to quality assessment on an Agilent Bioanalyzer 2100 system. The paired-end reads (150 bp) of the libraries were sequenced on the Illumina Hiseq 2500 platform (LC Sciences, USA). The original sequenced image data were converted into a sequence format by base calling to obtain FASTQ raw sequencing files.

### 4.4. Read Mapping and Analysis of Differential Gene Expression

The integrity of raw sequencing data sets was verified with md5 values. Raw reads from each sequencing library were first processed to filtered adaptor sequences, low-quality, and unknown reads using Trimmomatic v0.36 [58] with the following parameter settings: LEADING:3 TRAILING:3 SLIDINGWINDOW:4:15 and MINLEN:40, and finally clean reads for each sample were retained. The sheep reference genome sequence (Oar_v4.0) and the corresponding annotated transcript sets were directly downloaded from the sheep genome website (ftp://ftp.ncbi.nlm.nih.gov/genomes/all/GCA/000/298/735/GCA_000298735.2_Oar_v4.0). The HISAT2 v2.0.3 [59] program was employed to align the high-quality clean reads to the reference sequence, and indexes were created using Bowtie v2.2.3 [60] with default parameters. Non-normalized read counts for all detected genes were acquired by StringTie [61] with parameter settings -e -B -p 8, and a read count table was generated by the Python script “prepDE.py” in the StringTie package. The numbers of paired-end reads mapped to each gene were calculated and normalized to FPKM. Then, differential gene expression between any two samples was estimated with the DESeq2 [62] package in R (3.5.1). For differential expression analysis, an absolute value of log2 (fold change) ≥ 2 and an adjusted *p*-value ≤ 0.05 were set as thresholds for differential expression among DAF_1, DAF_2, and DAF_4. Because the gene expression profiles of the two cell types were similar, an absolute value of log2 (fold change) ≥ 1 and adjusted *p*-value ≤ 0.05 were set as thresholds for differential expression between DAFG5 and DAFG6. Further, analyses of gene expression patterns and KEGG pathways were conducted based on DEGs. All transcriptome analyses were performed on our own data analysis platform.

### 4.5. KEGG Pathway Enrichment and PPI Network Analysis

KEGG pathway analyses were performed with the DAVID program (http://david.abcc.ncifcrf.gov/) [63] and KOBAS program (http://kobas.cbi.pku.edu.cn/result_kobas.php?taskid=191111570271279) [64]. An adjusted *p*-value of less than 0.05 was designated as the threshold to determine significantly enriched KEGG pathway catalogs. Protein-protein association networks (PPI) analyses were performed with STRING V11 online (https://string-db.org/cgi/input.pl?sessionId=2oi9TlIogF0V&input_page_show_search=on) with the default parameters [65]. Data analyses and visualization were performed using our customized R scripts.

### 4.6. Quantitative Real-Time PCR (qPCR) Validation

Total RNA was isolated with RNAiso Plus (TAKARA, Beijing, China) according to the manufacturer’s instructions and checked with NanoDrop 2000c spectrophotometers (Thermo Scientific, Waltham, MA, USA). The cDNA was synthesized with the PrimeScript RT reagent kit (TAKARA, Beijing, China) according to the manufacturer’s instructions. Then, qPCR experiments were conducted on a LightCycler 480 II (Roche, Basel, Swiss) with TB Green Premix Ex Taq II (TAKARA, Beijing, China) and specific primers for *Fat-1*, *PRKACA*, *PPARA*, *FADS2,* and *ACACA* (Table S6), and the relative expression level of each gene was compared in R with the independent sample t-test method. A *p*-value < 0.05 was considered significantly different, and a *p*-value < 0.01 as extremely significantly different.

### 4.7. Determination of Polyunsaturated Fatty Acids

For polyunsaturated fatty acid content determination, 0.5 g of each gluteus muscle was collected and homogenized in 3 mL chloroform-methanol mixed medium. The supernatant was collected and then concentrated by N-EVAP. Then, 2 mL hexyl hydride was added to the residue, and after fully dissolving, 400 µl saturated KOH-methanol solution was added. The medium was placed at room temperature for 10 min after vigorous shaking for 2 min. Finally, the supernatant was collected and analyzed by GC-MS and then quantitated by external standard substances (Table S7).

## 5. Conclusions

In conclusion, the exogenous *Fat-1* gene originated from *C. elegans* and driven by the CAG promoter functioned well in our transgenic sheep and down-regulated the cell cycle pathway, *NF-κB* signaling pathway, *p53* signaling pathway, and *PI3K/Akt* signaling pathway, shifted the PUFA biosynthesis pathway toward high-level biosynthesis of *n*-3 LC-PUFAs, and finally increased *n*-3 LC-PUFA contents in the muscle.

## Data Availability

All the RNA-seq data used in this study were submitted to the National Center for Biotechnology Information (NCBI) Sequence Read Archive with the accession code PRJNA556979. The additional data supporting the conclusions in this paper can be found in the Supplementary Materials.

## Supplementary Materials

Supplementary materials can be found at https://www.mdpi.com/1422-0067/21/3/1121/s1.

## Abbreviations

DAF_1	Abnormal developed fetus at 100 gestational days
DAF_2	normal fetus at 100 gestational days
DAF_4	postnatal lambs
DAFG5	*Fat-1* transgenic-silencing skin cells
DAFG6	*Fat-1* transgenic-expressing skin cells
PUFAs	Polyunsaturated fatty acids
GMO	Genetically modified organism
DEG	Differentially expressed gene
FPKM	Fragments per kilobase of exon per million mapped reads
KEGG	Kyoto Encyclopedia of Genes and Genomes
PCA	Principal components analysis
PPI	Protein and protein interaction network
