# Supplementary material for: Comparative Transcriptome Analysis Provides Insights into the Polyunsaturated Fatty Acid Synthesis Regulation of Fat-1 Transgenic Sheep
Source: Int J Mol Sci. 2020 Feb 7;21(3):1121. doi: 10.3390/ijms21031121 (PMC7038019; doi:10.3390/ijms21031121)
Supplement: Supplementary file 1 [file ijms-21-01121-s001.zip › ijms-691108-supplementary-revised 1/ijms-691108-Supplementary Materials-revised.pdf]

Supplementary Figure legends：

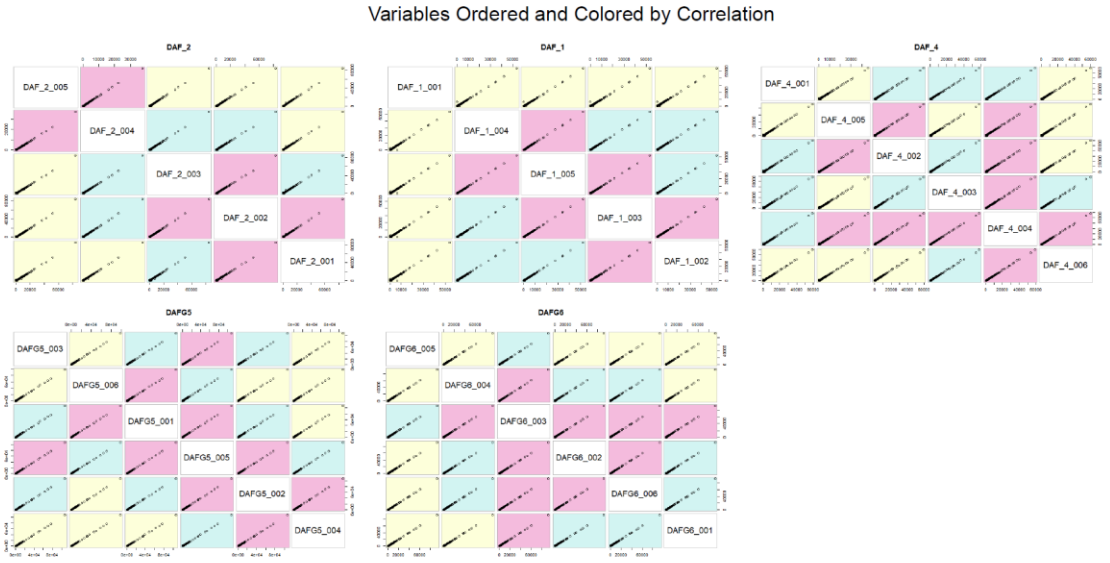

Figure S1. Variable ordered of sample pairs and colored by correlation. The biological repeatability of each sample was very reasonable and credible.

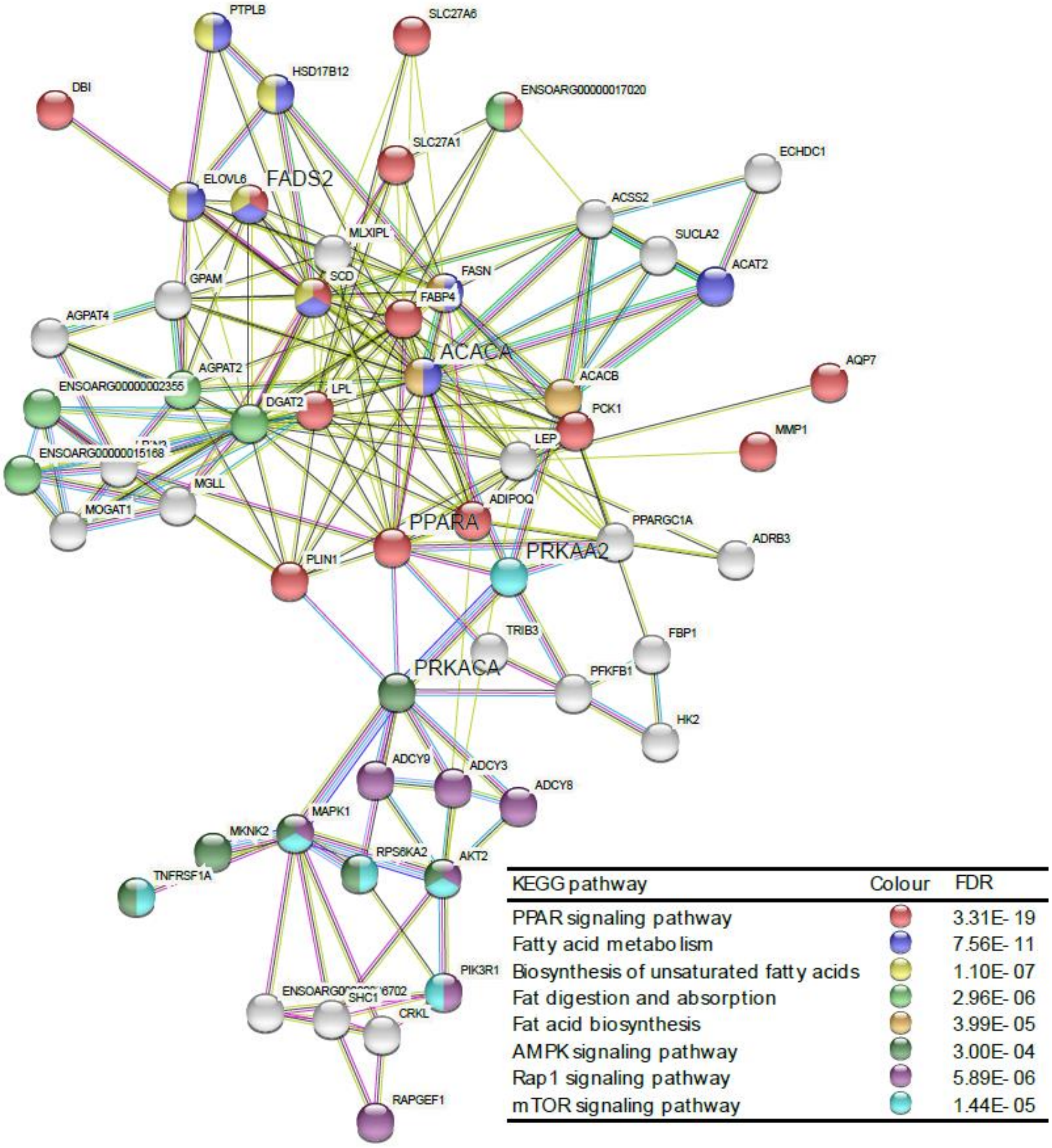

Figure S2. Protein and protein interaction network (PPI) for the genes associated with fatty acid biosynthesis and metabolism, cell proliferation and differentiation, and immunity. And key node genes have been tagged. Different colored balls represent different signaling pathways.

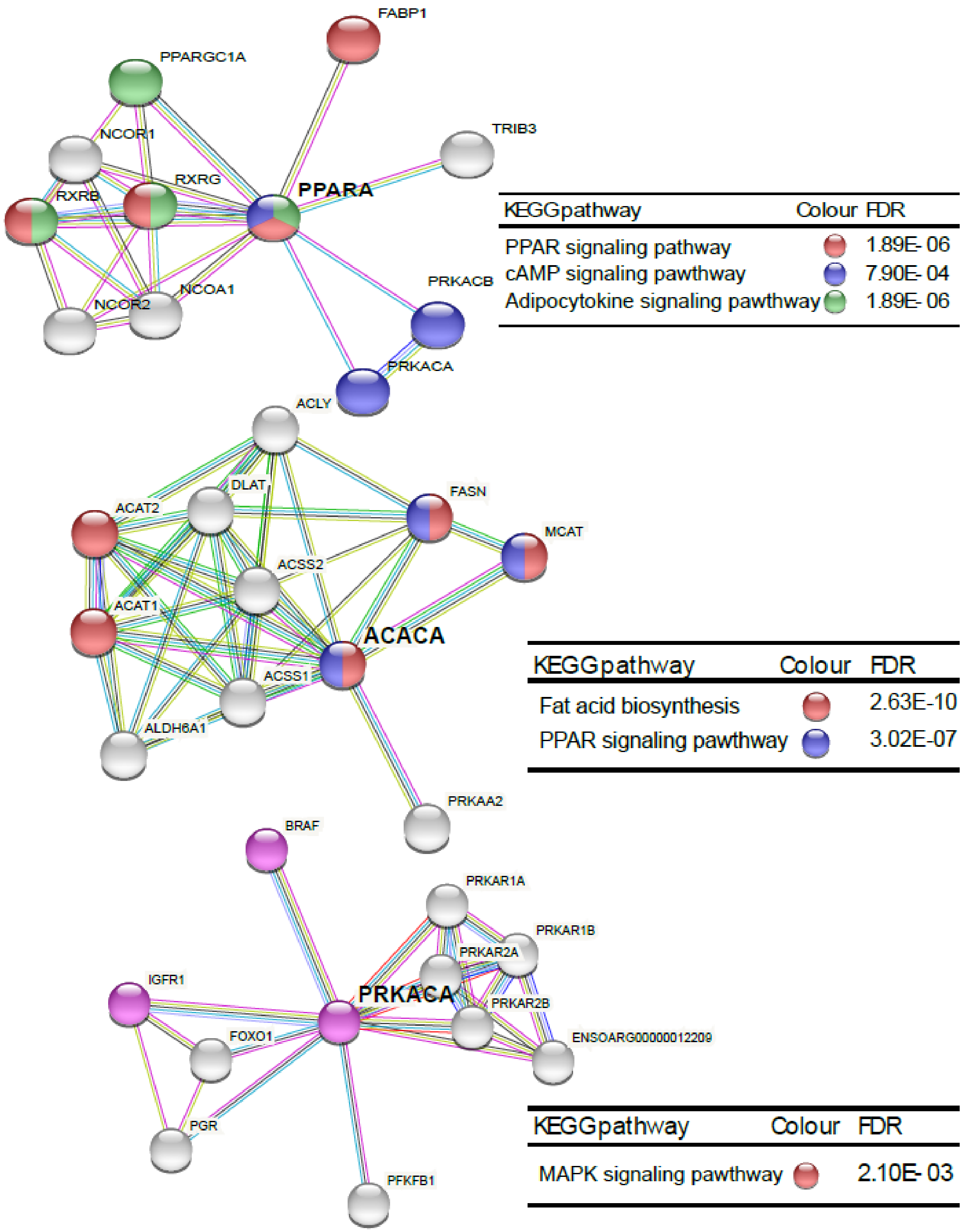

Figure S3. PPI network for PPARA, ACACA and PRKACA. PPARA involved in the PPAR signaling pathway, cAMP signaling pathway and Adipocytokine signaling pathway, which were related to fatty acid biosynthesis and metabolism; ACACA mainly involved in fatty acid

biosynthesis and PPAR signaling pathway; PRKACA mainly involved in MAPK signaling pathway.

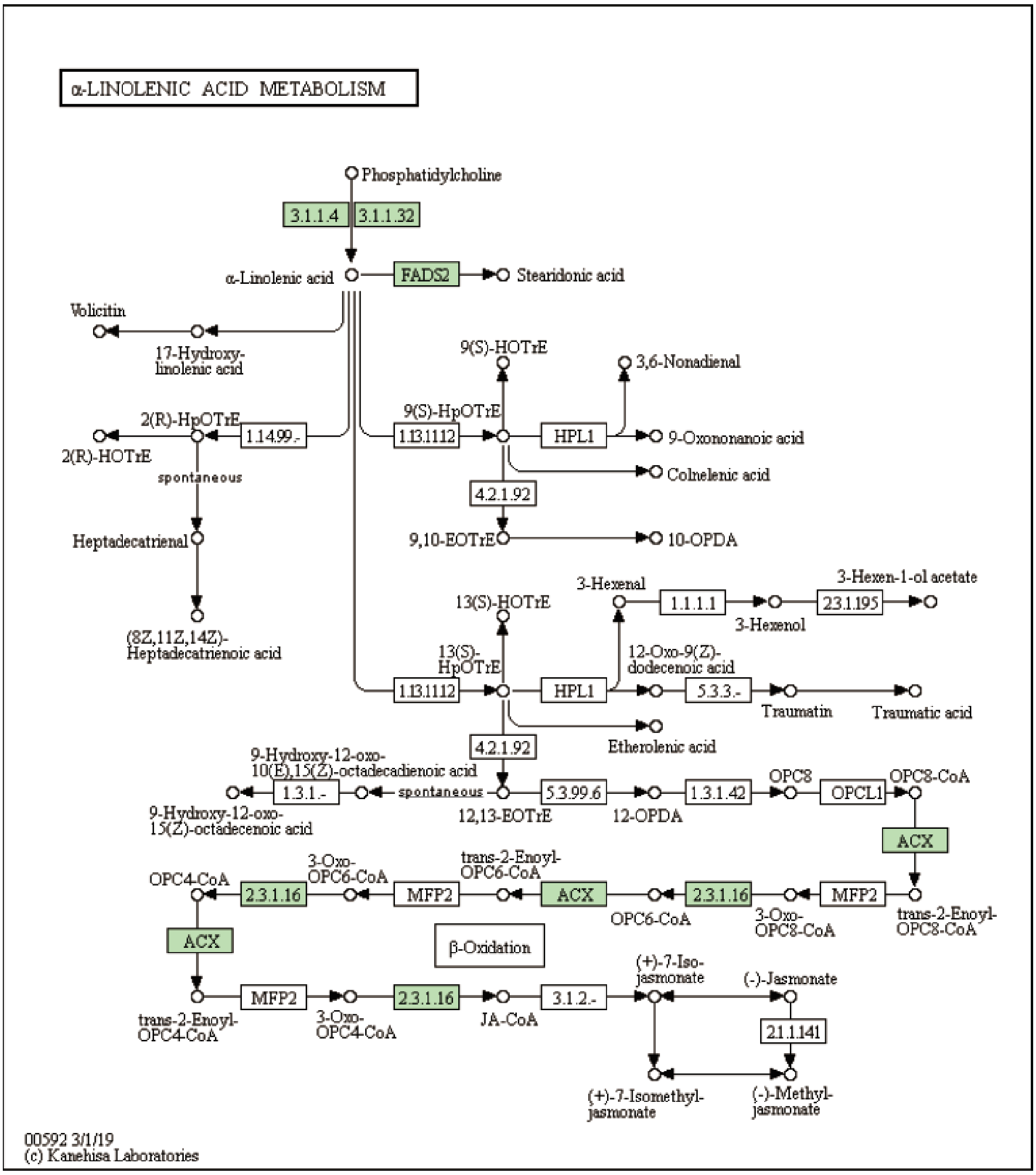

Figure S4. α-linolenic acid metabolism pathway.

Supplementary Table legends：

Table S1. The gene expression of Exogenous fat-1in ten tissues.

Table S2. Sample information and Sequencing statistics in this study.

Table S3. The basic alignment information for RNA-seq data generated from *Fat-1* transgenic sheep.

Table S4. The gene expression of all Ref. genes.

Table S5. The relative gene expression levels of key genes were validated by q-PCR.

Table S6. Specific primer sequences used in this study.

Table S7. Determination of fatty acid content.
